# Recipient vessel selection for microvascular chest wall reconstruction: A narrative review and decision support framework

**DOI:** 10.1016/j.jpra.2026.06.001

**Published:** 2026-06-13

**Authors:** Rushabh Shah, Chloe Jordan, Krzysztof Sosnowski, Mia Dogra, Christian Asher, Charles Malata

**Affiliations:** a Department of Plastic and Reconstructive Surgery, Addenbrooke’s Hospital, Cambridge, UK; b School of Clinical Medicine, University of Cambridge, Cambridge, UK; c Cambridge Breast Unit, Addenbrooke’s Hospital, Cambridge, UK; d Anglia Ruskin University School of Medicine, Cambridge & Chelmsford, UK

**Keywords:** Chest wall reconstruction, Microvascular, Free flap, Recipient vessels, Internal mammary vessels, Thoracodorsal vessels

## Abstract

**Background:**

Recipient vessel selection is a critical determinant of success in microvascular chest wall reconstruction. While the internal mammary and thoracodorsal vessels are most frequently used, prior surgery, radiotherapy, or flap design may necessitate alternative or salvage options. Despite its importance, no unified framework currently guides systematic vessel selection across reconstructive scenarios.

**Methods:**

A narrative review of the literature was conducted across PubMed, Medline, Embase, and Web of Science from database inception to August 2025. Studies reporting clinical outcomes, anatomical considerations, or vessel-specific complications in chest wall free flap reconstruction were included. Data were extracted from peer-reviewed studies, including case reports, case series, cohort studies, systematic reviews and meta-analyses.

**Results:**

Multiple recipient vessel systems are described, including the internal mammary, thoracodorsal, internal mammary perforator, thoracoacromial, lateral thoracic, circumflex scapular, intercostal, and cervical vessels, as well as vein grafts and arteriovenous loops. Each option demonstrates distinct advantages and limitations influenced by defect location, prior surgery or radiotherapy, pedicle requirements, preoperative assessment, and intraoperative vessel quality. Existing guidance on recipient vessel selection remains fragmented and is largely confined to breast reconstruction or institution-specific practice.

**Conclusion:**

This review summarises current evidence on recipient vessel selection in microvascular chest wall reconstruction and proposes a structured, evidence-informed decision-support framework. The framework is intended to aid preoperative planning and intraoperative strategy while recognising that final vessel choice must be tailored to individual patient factors and intraoperative findings.

## Introduction

Chest wall reconstruction poses significant challenges due to complex regional anatomy, respiratory mechanics, and the need to protect underlying intrathoracic organs. For large or composite defects following oncologic resection, trauma, or prior surgery, microvascular free tissue transfer may be required to provide durable soft tissue coverage and restore structural integrity.^1^, ^2^, ^3^, ^4^

Recipient vessel selection is a key determinant of success. The internal mammary (IMV), thoracodorsal (TDV), and internal mammary perforator (IMP) systems are most frequently used. However, prior surgery, radiotherapy, scarring, flap design, or vessel compromise may preclude their use.^5^^,^^6^ In such cases, thoracoacromial, lateral thoracic, circumflex scapular, intercostal, and cervical systems can serve as alternative options.

Most existing reviews focus predominantly on breast reconstruction, often comparing IMVs and TDVs, and do not address the broader anatomical or clinical challenges encountered in chest wall reconstruction.^7^^,^^8^ Furthermore, guidance on recipient vessel selection remains largely descriptive or context-specific, without an integrated framework that incorporates anatomical zones, preoperative assessment, prior interventions, and intraoperative vessel quality across the thoracic wall.

This narrative review synthesises the available evidence on recipient vessel selection for microvascular chest wall reconstruction, with a focus on anatomical considerations, surgical exposure, and reported clinical outcomes. Given the heterogeneity and predominantly low-level nature of the literature, the aim is to integrate existing data into a pragmatic, evidence-informed framework. The proposed framework is intended to support structured preoperative planning and intraoperative decision making in complex chest wall defects, rather than serve as a validated pathway.

## Methods

This study was conducted as a narrative review of the published literature on recipient vessel selection in microvascular chest wall reconstruction.

A literature search was performed across PubMed, MEDLINE, Embase, and Web of Science from database inception to August 2025 (Appendix 1). Eligible studies were peer-reviewed publications in English that reported the use of recipient vessels in microvascular free flap reconstruction of the chest wall. All clinical study designs were considered, including systematic reviews, meta-analyses, cohort studies, case series, and case reports. Reference lists of relevant articles were also reviewed to identify additional studies.

Studies were excluded if they were non-English, non-peer-reviewed, non-human studies, or were limited to flaps without microvascular anastomosis.

Included studies were reviewed narratively, with data extracted on recipient vessel anatomy, surgical exposure, vessel calibre, clinical outcomes, flap type where reported, complications, conversion to alternative recipient vessels, and reported advantages and limitations of each option. Given the heterogeneity of study design, defect location, flap choice, and outcome reporting, formal meta-analysis was not undertaken. Formal risk of bias assessment was also not performed, in keeping with the narrative review design.

The evidence was interpreted according to study design, sample size, clinical relevance, and consistency across reports. Where available, systematic reviews, meta-analyses, cohort studies, and larger clinical series were prioritised when developing the proposed framework. Smaller case series and case reports were used primarily to supplement anatomical, technical, or salvage reconstruction detail where higher-level evidence was limited. This information was synthesised comparatively and used to develop a zone-based, evidence-informed decision-support framework.

## Results

Multiple recipient vessel systems have been described, with the internal mammary (IMV), thoracodorsal (TDV), and internal mammary perforator (IMP) systems most frequently reported. The thoracoacromial, lateral thoracic, circumflex scapular, intercostal, and cervical vessels, as well as vein grafts and arteriovenous (AV) loops, are reported in previously treated or vessel-depleted fields.

**Fig. 1:**
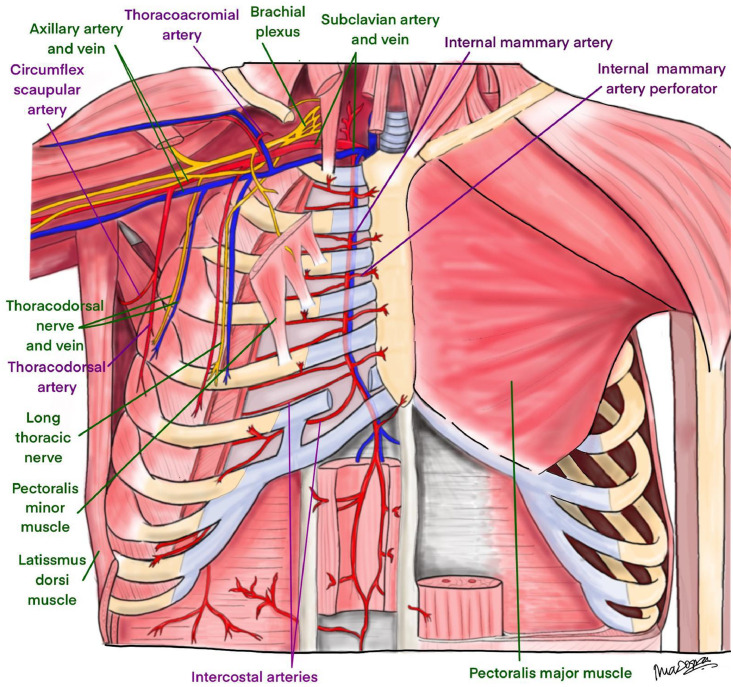
Anatomical illustration of major recipient vessels for chest wall free flap reconstruction, including the internal mammary system, thoracodorsal vessels, thoracoacromial branches, lateral thoracic vessels, circumflex scapular system, and intercostal vessels.

**Fig. 2:**
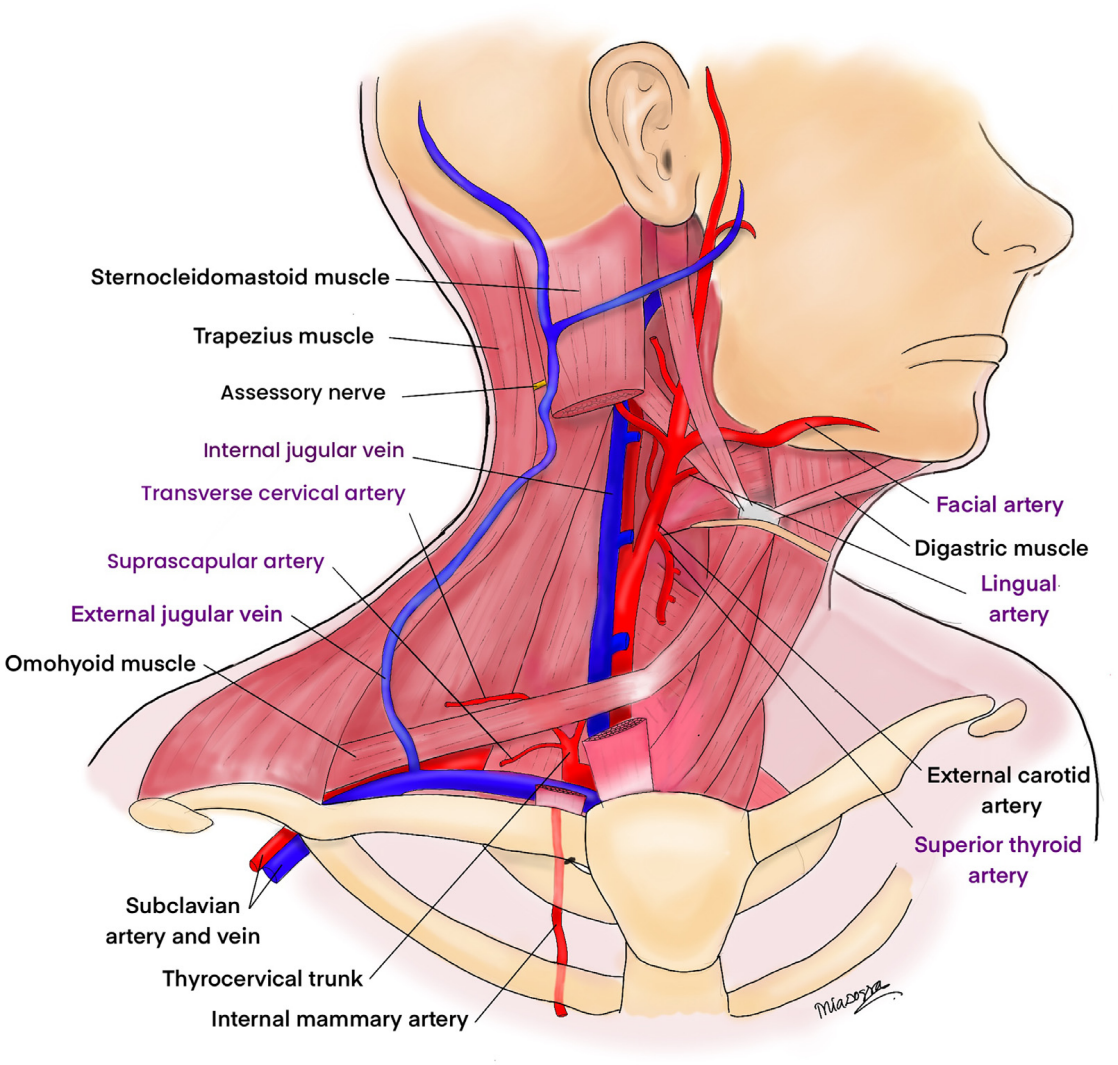
Cervical recipient vessels that may be used in chest wall reconstruction. The figure demonstrates superficial venous options, including the external jugular vein, and deeper recipient options including the internal jugular vein, transverse cervical and suprascapular vessels, and selected branches of the external carotid system.

**Fig. 3:**
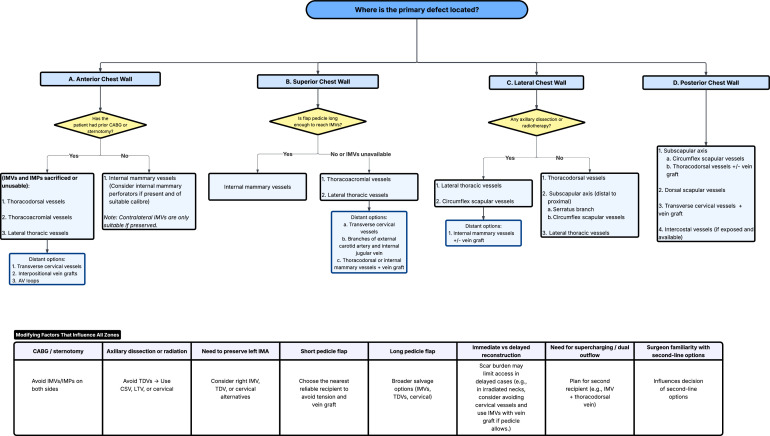
Proposed decision-support framework for recipient vessel selection in microvascular chest wall reconstruction. The framework is organised by defect location and key modifying factors affecting vessel choice. It is intended to support preoperative planning and intraoperative decision-making rather than serve as a fixed or validated algorithm. Prior operative history, radiotherapy, coronary artery bypass grafting or sternotomy, flap pedicle length, and available imaging should be reviewed before applying the framework. Where recipient vessel availability is uncertain, Doppler ultrasonography, computed tomography angiography, or other cross-sectional imaging may assist planning. Final recipient vessel choice should be confirmed intraoperatively based on vessel calibre, flow, quality, geometry, intimal integrity, size match, and pedicle reach.

The literature was synthesised to compare recipient vessels by calibre, exposure, indications, and outcomes. Figs. 1 and 2 show anatomical zones and vessel locations. Table 1 summarises anatomical characteristics, calibre, and access; Table 2 summarises outcomes and complications; Table 3 outlines existing frameworks. A zone-based, decision-support framework is then proposed to assist recipient vessel selection in complex chest wall reconstruction (Fig. 3).

**Table 1:** Recipient vessels used for microvascular chest wall reconstruction, summarising anatomical location, calibre, surgical access, and key reported advantages and limitations. Abbreviations: ICS, intercostal space; CTA, computer tomography angiography; LD, latissimus dorsi; TD, thoracodorsal; IMV, internal mammary vessel; TDV, thoracodorsal vessel; CABG, coronary artery bypass graft.

Recipient Vessel	Typical Calibre (mm)	Anatomical Location / Zone	Surgical Access	Key Advantages	Limitations / Considerations
**Internal Mammary**	Artery 2.4; Vein 2.5–3.0	1–2 cm lateral to sternum, deep to costal cartilages (parasternal / anterior zone)	2nd–3rd ICS incision, rib-sparing or limited cartilage resection	Central, consistent anatomy; good calibre; preserves TD axis	May require cartilage resection; pneumothorax risk; unavailable after CABG
**Internal Mammary Perforators**	Artery 1–1.9; Vein 1.5-3.0	Through intercostal/ pectoral muscle near sternal border (parasternal/ anterior zone)	Short parasternal incision, Doppler/ CTA-guided	Rib-sparing, superficial; preserves IMVs/TDVs	Variable anatomy; small calibre; challenging in obese or scarred fields
**Thoracodorsal**	Artery 1.8–2.0; Vein 2–4	Axillary region, branch of subscapular system (lateral zone)	Axillary incision, often during LD harvest	Reliable calibre; familiar exposure; short operative time	Reliability reduced after axillary dissection or radiation; lateral location limits medial inset
**Thoracoacromial**	Artery 1.0, Vein 1.5–3.0	Arises from axillary artery, deep to pectoralis minor (superior zone)	Infraclavicular incision under pectoralis major	Accessible for superior / clavicular defects; preserved after axillary surgery	Short pedicle; small calibre; technically deeper dissection
**Lateral Thoracic**	Artery 2.0, Vein 2.5–3.0	Lateral chest wall along serratus anterior (superior / lateral zone)	Axillary line or mastectomy incision	Simple access; preserved in immediate reconstructions	Anatomical variation; small calibre; fibrosis limits delayed use
**Circumflex Scapular**	Artery 2.5, Vein 3.0	Triangular space posterior to axilla (lateral / posterior zone)	Posterior axillary-fold incision	Good calibre; hidden scar; preserved after radiation	Technically demanding; moderate pedicle length; less suitable for anterior defects
**Intercostal**	Artery 1.0, Vein 1.5–2.0	Segmental vessels 4th–11th ICS (posterior zone)	Along ribs or resection margins	Useful salvage for posterior / lateral defects; predictable anatomy	Short pedicle; pleural injury risk; high technical difficulty
**Cervical**	Artery 2–3, Vein 3–4	Neck region (superior zone)	Neck extension or supraclavicular incision	Large calibre; preserved after thoracic surgery	Distant from chest wall; visible scar; may require vein graft
**Vein Grafts / AV Loops**	Vein 3	Custom to defect	Vein harvest (deltopectoral groove / thigh); loop construction	Extends pedicle reach; restores flow in vessel-depleted fields	Added operative time; donor-site morbidity; higher thrombosis risk

**Table 2:** Comparative outcomes of recipient vessels in microvascular chest wall reconstruction. Abbreviations: STA, superior thyroid artery; TCV, transverse cervical vessel; EJV, external jugular vein; CABG, coronary artery bypass grafting.

Recipient Vessel	Reported Flap Survival (%)	Typical Complications	Representative References	Key Clinical Notes
**Internal Mammary**	98–99	Pneumothorax < 1.5%; rare thrombosis	Saint-Cyr 2007; Lemdani 2024; Rozen 2013	Reliable first-line recipient for medial defects; robust calibre; avoid after CABG
**Internal Mammary Perforators**	97–100	3–5% fat necrosis; occasional conversion	Saint-Cyr 2007; Fernandez-Diaz 2019	Rib-sparing option; 35–40% anatomical suitability; useful in irradiated fields
**Thoracodorsal**	97–99	Seroma 12%; reduced patency in irradiated axilla	Samargandi 2017; Rozen 2013	Preferred for lateral defects; avoid if prior axillary dissection or radiation
**Thoracoacromial**	95–100 (small series)	Minimal; occasional wound dehiscence	Singh 2017; Kim 2019	Accessible for short-pedicle flaps and superior defects
**Lateral Thoracic**	100	No reported vascular events	Fong 2016; Muto 2023	Useful in immediate cases with preserved axillary vessels; variable anatomy
**Circumflex Scapular**	98–100	Minor venous congestion or dehiscence	Lantieri 1999; Santanelli 2015	Good calibre; favoured for posterior/lateral defects; spared by radiation
**Intercostal**	≈ 85–90 (small series, salvage cases)	Pleural injury; challenging dissection	Duteille 2004; Wei 2004	Consider for posterior salvage when local vessels unavailable
**Cervical**	≈ 100	Minor seroma; rare flap loss	Banic 1995; Mehrara 2003; Bigdeli 2024	Reliable inflow/outflow in vessel-depleted fields; distance may require grafting
**Vein Grafts / AV Loops**	95–100 (planned use); lower and variable in salvage scenarios	Thrombosis; wound breakdown	Engel 2007; Nelson 2015; Chuong 2023	Extends pedicle reach; effective in depleted fields but adds operative time

**Table 3:** Published frameworks for recipient vessel selection in microvascular chest wall and breast reconstruction, summarising clinical context, preferred and alternative recipients, and key distinguishing features or limitations.

Author (Year)	Clinical Context	Primary Recipient	Alternative Recipient	Key Features / Limitations
**Hamdi et al. (2004)**	Breast reconstruction using rib-sparing internal mammary perforators	IMPs	IMVs → TDVs	Early rib-sparing technique; excellent for perforator flaps but not generalisable to non-perforator or salvage settings.
**Ramakrishnan et al. (2013)**	Institutional breast reconstruction pathway	TDVs	IMVs or IMPs	Reflects institutional preference and surgeon familiarity; highlights influence of local experience on vessel choice.
**Samargandi et al. (2017)**	Systematic review assessing comparative outcomes of IMVs and TDVs	IMVs or TDVs (equivalent)	—	Evidence-based equivalence between IMV and TDV systems; supports surgeon discretion according to exposure and defect.
**Ono et al. (2013)**	Post-axillary clearance or irradiated axilla	IMVs	IMPs or CSVs	Prioritises IMVs when TDVs compromised; aims to preserve latissimus dorsi; limited by single-centre sample size.
**Bigdeli et al. (2024)**	Sternal / anterior chest wall reconstruction after cardiac surgery	IMVs	Cephalic vein turndown or AV loop	Focused on post-sternotomy reconstruction; practical algorithm for vessel-depleted mediastinal fields.

### Internal mammary vessels

The IMVs course parasternal, arising from the subclavian system and draining into the brachiocephalic vein with a mean arterial calibre of 2.4 mm and venous calibre of 2.5–3.0 mm. Exposure is achieved at the second or third intercostal space, either by cartilage removal or rib-sparing dissection to minimise morbidity.^9^^,^^10^

IMVs are the most frequently reported recipient vessels in microvascular chest wall reconstruction, with several case series documenting flap survival rates of 98–99% and conversion rates (to another recipient) below 2%.^11^^,^^12^ O’Neill et al. reported 99.5% flap success with a < 2% conversion rate, even in irradiated fields.^11^ Although IMVs are generally reliable, their use is associated with specific exposure-related considerations. Cartilage resection, where required, may increase morbidity, including pneumothorax, chest wall contour deformity, and pain. Rates of pneumothorax are reported to occur in <1.5%, and venous thrombosis or haemothorax account for flap failure rates consistently under 2%.^7^^,^^13^, ^14^, ^15^ Comparative data show lower fat-necrosis rates in breast reconstruction when using IMVs compared with thoracodorsal vessels (12%vs 30%, P < 0.05).^16^ Their consistent anatomy and vessel calibre make them reliable first-line recipients for medial and parasternal defects.^17^^,^^18^ Reported limitations include the need for limited cartilage resection in selected cases and loss of availability after coronary artery bypass grafting (CABG).^19^, ^20^, ^21^, ^22^ Additionally, they may be absent due to sacrifice during tumour ablation or radical tissue debridement.

### Internal mammary perforators (IMPs)

IMPs arise from IMVs, most commonly in the second or third intercostal space, and course through the intercostal and pectoral musculature to the subcutaneous plane.^23^ Typical arterial and venous calibres range from 1.0–1.9 mm and 1.4–2.9 mm, respectively.^24^ They are localised pre- or intraoperatively using Doppler ultrasonography; their superficial position allows exposure through a short parasternal incision without rib or cartilage resection.^25^^,^^26^

A 2023 meta-analysis of 313 flaps reported a pooled success rate of 99.8% and an overall complication rate of 11%, with vascular complications (5%) and fat necrosis (3%) most frequent.^24^ Outcomes are comparable to those achieved with internal mammary vessels, including in irradiated fields where IMPs remain accessible due to their superficial course.^7^ Kropf et al. observed no significant difference in flap loss between IMPs and IMVs in 158 patients.^16^

Their main limitation is anatomical variability, with large case series reporting suitability rates of only 35–40%.^7^^,^^16^ This is due to absence, size mismatch, or prior surgical disruption. Additional technical challenges include difficult dissection in high-BMI or scarred fields and small venous calibre, which may predispose to thrombosis.^27^ These findings indicate that while IMPs provide a reliable rib- and muscle-sparing recipient option, their use depends on intraoperative assessment of vessel presence and quality.

### Thoracodorsal vessels (TDVs)

The thoracodorsal artery arises from the subscapular artery and runs along the lateral chest wall, accompanied by venae comitantes that drain into the axillary vein. The vessels are typically accessed through the axilla, either during latissimus dorsi harvest or via a separate incision. Mean arterial diameter ranges from 1.8–2.0 mm and venous diameter from 2–4 mm, providing a close match for most free flap vascular pedicles.

TDVs are among the most frequently used recipient vessels in microvascular chest wall reconstruction, with reported flap survival rates of 97–99% across multiple studies.^28^^,^^29^ Gravvanis et al. (2008) documented a 97.5% flap survival rate in delayed breast reconstructions using TDVs.^29^ In a meta-analysis by Samargandi et al. (2017), no statistically significant difference in flap loss was found when comparing TDVs to IMVs.^12^ Operative and anastomotic times were frequently shorter than with IMVs, particularly when TDVs were already exposed during concurrent axillary surgery.^26^

However, usability may be reduced in irradiated or previously dissected axillae due to fibrosis, vessel fragility, or obliteration of the vascular pedicle. Temple et al. (2005) reported that TDVs were unusable in up to 26% of delayed or irradiated cases, necessitating intraoperative conversion to alternative recipients.^30^ Reported complications include seroma and lymphoedema related to axillary lymphatic disruption, but overall flap survival remains consistently high.^29^

### Thoracoacromial vessels (TAVs)

The thoracoacromial artery arises from the second part of the axillary artery and divides into acromial, clavicular, deltoid, and pectoral branches that lie deep to pectoralis minor.^31^ The pectoral branch is most frequently used and demonstrates limited anatomical variation.^32^ Exposure is achieved through an infraclavicular or upper chest wall incision with sub-pectoral dissection to identify the pedicle. Sacrifice of the pectoral branch carries minimal morbidity due to robust collateral supply.^33^ The exposure field is narrower than for IMV or TDV because of the deeper course.

TAVs have been employed in delayed breast, mediastinal, and head-and-neck reconstructions, particularly when IMV or TDV are unavailable or when bipedicled or short-pedicle flaps are used.^34^, ^35^, ^36^ Small series report 100% flap survival in 3–6 cases without major complications.^37^^,^^38^ Kompatscher et al. (2005) found the thoracoacromial pedicle consistently present and technically reliable in salvage reconstructions.^32^ Limitations include a relatively deep operative field, shorter pedicle length, and smaller calibre compared with primary thoracic recipient vessels. Mean arterial calibre is approximately 1.0 mm and venous calibre 1.5–3.0 mm.^39^, ^40^, ^41^

### Lateral thoracic vessels (LTVs)

The lateral thoracic artery most commonly originates from the second part of the axillary artery and courses along the lateral border of pectoralis minor over the serratus anterior.^42^ Anatomical variation is frequent, with duplication, hypoplasia, or variable origins reported in up to 50% of patients.^42^ The vessels can be accessed through an axillary or mastectomy incision, or along the lateral chest wall.^43^ Pedicle length typically ranges from 4–6 cm, though proximal dissection toward the axillary artery may be required to gain additional reach.

LTVs have been used successfully in immediate breast reconstruction when preserved during axillary surgery.^44^^,^^45^ Fong et al. (2016) reported no flap losses or vascular complications in four cases using LTVs,^46^ while Muto et al. (2023) described 100% flap survival in seven cases without conversion or anastomotic difficulties, highlighting their role when TDV have been sacrificed.^47^ The arterial calibre averages 2.0 mm and venous calibre 2.5–3.0 mm, providing a good match for smaller or short-pedicle flaps.^42^

Although technically straightforward to expose, their reliability depends on preservation during axillary dissection or clearance, whilst scarring or fibrosis may reduce suitability in delayed reconstructions.^47^ Reported usability in previously operated axillae is therefore variable across series.^44^, ^45^, ^46^, ^47^

### Circumflex scapular vessels (CSVs)

The circumflex scapular artery arises as the first branch of the subscapular artery and courses posteriorly through the triangular space bordered by the long head of triceps, teres major, and subscapularis.^6^^,^^48^ It is accompanied by one or two venae comitantes and gives off small muscular branches deep to teres minor. Exposure is achieved via an incision along the posterior axillary fold or scapular region, identifying the pedicle as it exits the triangular space. Pedicle length typically ranges from 6–8 cm, and the anastomosis is usually performed laterally on the chest wall, allowing the scar to be concealed within the axillary fold.^48^

CSVs have been reported as reliable recipient vessels, particularly when TDVs are unavailable after axillary clearance or irradiation, and IMVs are inaccessible. Lantieri et al. (1999) described 40 cases of autologous breast and chest wall reconstruction using CSVs with no flap loss and a 27.3% complication rate, mainly due to wound breakdown or venous congestion.^49^ Additional series have confirmed preserved vessel calibre and flow characteristics even in vessel-depleted fields.^50^^,^^51^ The artery and vein measure approximately 2.5 mm and 3.0 mm in diameter respectively, providing a good calibre match for most free flaps. Reported challenges include moderate pedicle length and more complex orientation for medial chest wall inset.^48^

### Intercostal vessels

The intercostal vessels arise segmentally from the thoracic aorta and the internal mammary system, with perforators present from approximately the fourth to eleventh intercostal spaces.^52^ Their anatomy is generally consistent, although pedicle length is short and exposure requires careful dissection within the intercostal space.^53^^,^^54^ Perforator location determines flap classification, with lateral perforators arising from the costal segment, anterior perforators from the muscular or rectus segments, and dorsal perforators from the vertebral segment.^53^

These vessels are most often encountered opportunistically when ribs are resected for tumour or infection, allowing the cut vessel ends to be mobilised for microvascular anastomosis. Intentional exposure is technically demanding and carries a risk of pleural injury.

Clinical experience with intercostal vessels as recipient sites remains limited to small series and case reports.^53^, ^54^, ^55^, ^56^, ^57^ They have been used primarily for salvage or secondary free flap reconstruction of posterior or lateral chest wall defects.^46^^,^^47^ Hamdi et al. (2008) reported flap survival in seven of eight cases using intercostal vessels, with one early failure from venous thrombosis, while Duteille et al. (2020) described 100% success in five cases using intercostal perforators with minimal complications and stable long-term results.^53^^,^^55^ Reported complication rates across studies are low,^55^^,^^56^ but short pedicle length and technical difficulty restrict wider adoption.

### Cervical vessels

The most common neck recipient vessels include branches of the thyrocervical trunk and external carotid artery, such as the transverse cervical and superior thyroid arteries.^58^ Venous drainage is typically achieved via the external or internal jugular veins. These vessels can be accessed through supraclavicular or extended chest wall incisions; the external jugular vein is readily exposed, while the transverse cervical artery is located along the lateral neck at the level of the clavicle. Arterial calibre averages 2–3 mm, and venous calibre averages 3–4 mm.^59^^,^^60^

Cervical vessels are primarily utilised in complex reconstructions or vessel-depleted fields, such as after sternectomy, mediastinal infection, or prior flap failure. Bigdeli et al. (2024) described a stepwise algorithm in which cervical vessels served as preferred salvage inflow, reporting 100% flap survival in 15 patients when combined with vein grafts.^61^ Sauerbier et al. (2011) demonstrated the feasibility of supercharging rectus and anterolateral thigh flaps to the superior thyroid artery and internal jugular system without vascular complications.^62^ Mehrara et al. (2003) observed that jugular-vein augmentation of venous outflow reduced congestion in large or bipedicled flaps.^63^

Although cervical vessels offer dependable calibre and anatomy and are often preserved following thoracic surgery or radiotherapy, their use may necessitate vein grafts due to distance from the chest wall, and visible cervical scarring can be a cosmetic limitation.

### Vein grafts and AV loops

Vein grafts and arteriovenous (AV) loops are established salvage strategies when local recipient vessels are unavailable or of inadequate length or calibre. The cephalic vein is the most commonly used conduit owing to its consistent anatomy, favourable calibre (∼3 mm), and reliable course along the deltopectoral groove. Alternative options include the great and short saphenous veins.^64^^,^^65^

Exposure typically involves an incision along the deltopectoral groove for cephalic harvest, after which the vein may be used as an interposition graft to extend pedicle length or configured as an AV loop. In the latter approach, the vein is first anastomosed to a suitable donor artery and vein to create a temporary circuit near the defect, which is subsequently divided for definitive flap anastomosis.^66^ The saphenous veins may similarly be used for pedicle extension in chest wall reconstruction.^67^

Clinical reports describe high flap survival when these techniques are used in vessel-depleted or irradiated fields. Rahman et al. (2020) and Chuong et al. (2023) each reported 94–100% flap success in such series.^68^^,^^69^ These methods are particularly utilised in sternal reconstruction, delayed flap transfers, and salvage following vascular thrombosis. The cephalic-subclavian venous system provides favourable high-flow and low-pressure haemodynamics, contributing to reliable outcomes. Despite their success, vein grafts and AV loops add operative time and require an additional donor site, with a slightly higher risk of thrombosis compared with direct anastomosis.^70^ They should therefore be considered salvage or planned adjunctive strategies rather than routine first-line recipient options.

### Interpretation of reported outcomes

Reported flap survival rates were generally high across recipient vessel systems; however, these figures should be interpreted with caution. Much of the available evidence consists of retrospective studies, small case series, and selected reports of successful reconstruction, which may introduce publication bias and overestimation of real-world success. Studies also varied substantially in defect location, flap type, prior treatment, recipient vessel exposure, and reporting of complications. In particular, thrombosis, intraoperative conversion to alternative recipients, vessel usability, and failure of planned recipient selection were inconsistently reported. For this reason, reported survival rates should be interpreted alongside technical feasibility, anatomical reliability, pedicle reach, vessel quality, and suitability in irradiated or previously operated fields.

### Existing decision-making frameworks

Published frameworks for recipient vessel selection remain largely context-specific. They focus on perforator-based breast reconstruction, institution-led pathways, irradiated axillae, or post-sternotomy salvage, and do not provide a cross-thoracic solution. A comparative summary of the most cited models, including clinical scope, preferred and alternative recipients, and limitations, is presented in Table 3.

## Discussion

Recipient vessel selection is a critical determinant of success in microvascular chest wall reconstruction, yet practice remains heterogeneous across institutions and clinical contexts. Although the IMV and TDV systems are well described, the majority of the literature remains descriptive, focusing on anatomy or exposure techniques rather than structured decision-making. Consequently, vessel choice is often guided by surgeon preference or institutional practice. Based on the anatomical and clinical evidence collated in this review, we developed an evidence-informed, zone-based decision-support framework (Fig. 3) to support systematic recipient vessel selection in microvascular thoracic reconstruction.

Preoperative assessment is an important first step before applying this framework. Previous operative notes, radiotherapy history, planned oncological resection, prior coronary revascularisation, and available cross-sectional imaging should be reviewed. Where recipient vessel availability is uncertain, computed tomography angiography (CTA), Doppler ultrasonography, or other imaging modalities may help assess vessel patency, calibre, location, and the presence of suitable perforators or venous conduits.^13^**^,^**^25^**^,^**^26^ This is particularly relevant in previously operated, irradiated, scarred, or vessel-depleted fields. However, imaging should be regarded as an adjunct to operative planning rather than a substitute for intraoperative vessel assessment.

The proposed framework is organised into four anatomical zones: parasternal/anterior, superior, lateral, and posterior, with defined escalation pathways based on defect location, pedicle reach, prior treatment, and intraoperative vessel quality. Distant options, including cervical vessels and vein grafts or arteriovenous loops, are incorporated as contingency branches, while cross-cutting modifiers such as previous coronary artery bypass grafting, axillary clearance, and the need to preserve the left internal mammary artery are applicable across all zones. Supporting evidence for each branch is summarised in Table 4.

**Table 4:** Evidence base supporting key decision nodes in the proposed recipient vessel selection decision-support framework for microvascular chest wall reconstruction. Abbreviations: IMV, internal mammary vessel; IMP, internal mammary perforator; TDV, thoracodorsal vessel; TAV, thoracoacromial vessel; LTV, lateral thoracic vessel; CSV, circumflex scapular vessel; EJV, external jugular vein; CABG, coronary artery bypass grafting.

Decision Node	Primary Strategy	Key Supporting References	Evidence Summary / Rationale	Evidence Type
**Anterior Zone**	Use IMVs or IMPs if available; avoid after CABG / sternotomy	O’Neill 2011; Saint-Cyr 2007	IMVs provide reliable calibre and central location; prior sternotomy or CABG sacrifices the internal mammary axis	Large case series; cohort studies
**Superior Zone**	Prefer IMVs; if reach limited use TAV / LTV; escalate to cervical if needed	Kompatscher 2005; Kim 2019; Banic 1995	IMVs accessible with long pedicles; subclavicular vessels provide viable regional alternatives	Large case series; cohort studies
**Lateral Zone**	Use TDVs; if unsuitable use CSV → LTV → cervical	Samargandi 2017; Temple 2005; Ono 2013; Lhuaire 2017	TDVs reliable in virgin axilla; radiation or dissection reduces usability; CSV preserved after axillary treatment	Meta-analysis; cohort studies; large case series
**Posterior Zone**	Use CSVs; if inadequate calibre, consider TDV branch or intercostals	Lantieri 1999; Santanelli 2015; Sharp 2021; Otani 2020	CSV offers consistent calibre and posterior exposure; intercostals limited by short pedicle and technical difficulty	Case series; case reports
**Prior CABG / Sternotomy**	Avoid IMVs / IMPs; use TDV or cervical inflow	Bigdeli 2024	CABG or sternotomy sacrifices IMVs; cervical options maintain reliable inflow	Retrospective case series
**Axillary Dissection / Radiation**	Avoid TDVs; use LTV, CSV or IMVs depending on reach	Ono 2013; Lhuaire 2017; Temple 2005	Axillary fibrosis compromises subscapular axis; lateral or cervical systems remain patent	Retrospective case series
**Pedicle Length / Reach**	Match nearest reliable recipient for short pedicles; long pedicles allow cross-zonal reach	Saint-Cyr 2007; Temple 2005	Short pedicles (TUG, gracilis) require nearby recipients; long pedicles (ALT, LD) extend reach	Large case series; cohort studies
**Intra-operative Vessel Quality**	Escalate to next-tier or distant recipient if vessel scarred or fibrotic	Rozen 2013; Saint-Cyr 2007	Poor flow or mismatch necessitates intra-operative adaptability	Large case series; cohort studies
**Venous Outflow / Supercharging**	Plan auxiliary drainage via EJV or contralateral IMV for large flaps	Hwee 2020; Nelson 2015; Lantieri 1999	Dual venous drainage reduces congestion and improves reliability	Case series; case reports

For anterior and parasternal defects, the IMV system remains the most established and reliable recipient option, demonstrating consistently high flap survival and low complication rates even in irradiated fields.^7^^,^^11^, ^12^, ^13^, ^14^, ^15^ Comparative analyses show lower fat-necrosis rates with IMVs than TDVs (12%vs 30%, P < 0.05).^16^ Internal mammary perforators provide a rib-sparing alternative where anatomy permits, although suitability is limited by perforator calibre and consistency, particularly in obese or scarred patients.^7^^,^^16^^,^^24^^,^^27^ Where prior sternectomy or CABG renders the internal mammary axis unusable, nearby thoracic recipients should be considered to minimise unnecessary dissection distant from the defect. In this context, the lateral thoracic vessels may be preferred over CSVs for anterior defects, with choice determined by pedicle reach and vessel quality. If no suitable thoracic option is available, cervical inflow or vein-graft/AV-loop strategies provide dependable salvage.^71^

Superior or clavicular defects may be reconstructed using the IMV when pedicle length allows. When reach is inadequate or the system has been sacrificed, subclavicular options such as the thoracoacromial or lateral thoracic vessels provide effective regional alternatives with acceptable calibre and accessibility.^31^^,^^32^^,^^37^ Cervical vessels serve as dependable salvage recipients when regional options are unsuitable, although in this framework they are prioritised after local subclavicular systems to limit the need for interposition grafting and reduce thrombosis risk.^61^, ^62^, ^63^^,^^70^

For lateral and axillary defects, the TDV system is typically first line owing to consistent anatomy and straightforward exposure, with comparable flap survival to internal mammary recipients on meta-analysis.^12^^,^^28^, ^29^, ^72^ However, its reliability diminishes following axillary dissection or radiotherapy, necessitating alternative strategies.^29^ In vessel-depleted axillae, preserved lateral thoracic or circumflex scapular vessels are prioritised, with internal mammary or cervical inflow reserved as distant options, often requiring vein grafting depending on pedicle length.^11^^,^^12^^,^^42^, ^43^, ^44^, ^45^, ^46^, ^47^^,^^49^, ^50^, ^51^^,^^70^

Posterior thoracic and paraspinal reconstructions rely primarily on the circumflex scapular system because of its posterior orientation, consistent calibre, and preserved flow even in irradiated fields.^48^, ^49^, ^50^, ^51^ Intercostal vessels can be used selectively for posterior or lateral salvage, although their short pedicle and proximity to the pleura increase technical complexity.^46^^,^^47^^,^^53^^,^^54^ Where circumflex scapular orientation or calibre is suboptimal, posterior mobilisation of the thoracodorsal or serratus branch may allow primary anastomosis at the expense of additional dissection.^49^, ^50^, ^51^ Cervical inflow remains a less favourable but viable option for select high posterior defects.^61^, ^62^, ^63^

When no suitable thoracic recipient is available, cervical vessels and vein grafts or arteriovenous loops provide reliable salvage pathways in vessel-depleted or redo reconstructions.^61^^,^^68^^,^^69^ Although associated with longer operative times and an increased thrombosis risk, these strategies preserve reconstructive feasibility in complex cases and are therefore incorporated into all algorithmic branches as contingency options.^64^, ^65^, ^66^, ^67^^,^^70^

Several modifying factors influence recipient suitability across all zones. Prior sternotomy or coronary bypass mandates avoidance of IMV, while axillary dissection or radiotherapy reduces TDV reliability.^11^^,^^29^ Pedicle length remains a key determinant of proximity-based selection, with short-pedicle flaps favouring local recipients and longer pedicles allowing cross-zonal flexibility.^17^^,^^18^ Preservation of the left internal mammary artery is recommended in patients at cardiac risk, and dual venous outflow may be considered for large or bipedicled flaps to mitigate venous congestion.^63^ Recognising these modifying factors enables intraoperative adaptability without compromising reconstructive outcomes.

Intraoperative findings remain decisive. Even when a recipient vessel is favoured by anatomical zone or preoperative imaging, poor flow, scarring, intimal damage, friability, inadequate calibre, size mismatch, or unfavourable geometry may require conversion to an alternative recipient vessel or use of a vein graft or arteriovenous loop. The proposed framework should therefore be interpreted as a structured planning aid that supports contingency planning, rather than as a fixed sequence that overrides surgical judgement.

Previous algorithms have provided valuable insights but are typically limited to specific clinical contexts such as breast reconstruction, irradiated axillae, or post-cardiac salvage.^12^^,^^61^^,^^71^^,^^73^^,^^74^ The present framework expands upon these by integrating zone-based anatomical mapping with conditional decision logic applicable across the thoracic wall, translating heterogeneous evidence into a practical decision-support tool.

This review has several limitations. The underlying evidence is largely retrospective, with heterogeneous reporting of vessel usability, flap type, complications, and outcomes, limiting comparative statistical analysis. Reported flap survival rates are consistently high but may overestimate real-world performance because of publication bias and selective reporting of successful reconstructions. The proposed framework is evidence-informed rather than prospectively validated and remains dependent on surgeon experience, anatomical familiarity, available imaging, and institutional resources. It should therefore be regarded as a supportive decision-making aid rather than a definitive or validated pathway. Future studies incorporating prospective multicentre data, standardised reporting of recipient vessel usability, and validation of decision frameworks would help refine vessel selection in complex chest wall reconstruction.

## Conclusion

Recipient vessel selection in microvascular chest wall reconstruction requires a structured yet adaptable approach that accounts for patient factors, defect characteristics, prior treatment, preoperative assessment, and intraoperative vessel quality. The proposed zone-based evidence-informed framework integrates anatomical, technical, and clinical considerations into a practical decision-support tool to aid preoperative planning and intraoperative strategy. Given the heterogeneity of the underlying evidence, this framework should be interpreted as a supportive guide rather than a definitive pathway, with final decision-making tailored to individual patient characteristics and intraoperative findings.

## Funding

None.

## Ethical approval

Not required.

## Conflicts of interest

None declared.

## Appendix Supplementary materials

Image, application 1
