# Supplementary material for: Recipient vessel selection for microvascular chest wall reconstruction: A narrative review and decision support framework
Source: JPRAS Open. 2026 Jun 13;51:252–68. doi: 10.1016/j.jpra.2026.06.001 (PMC13382177; doi:10.1016/j.jpra.2026.06.001)
Supplement: Supplementary file 1 [file mmc1.docx]

***Appendix 1***

**EMBASE via OVID**Ovid MEDLINE(R) and Epub Ahead of Print, In-Process, In-Data-Review & Other Non-Indexed Citations, Daily and Versions <1946 to June 24, 2025>

1 ('chest wall reconstruction' or 'breast reconstruction' or 'anterior chest wall').mp. [mp=title, book title, abstract, original title, name of substance word, subject heading word, floating sub-heading word, keyword heading word, organism supplementary concept word, protocol supplementary concept word, rare disease supplementary concept word, unique identifier, synonyms, population supplementary concept word, anatomy supplementary concept word] 16279

2 ('recipient vessel' or 'recipient site' or 'microvascular anastomosis').mp. [mp=title, book title, abstract, original title, name of substance word, subject heading word, floating sub-heading word, keyword heading word, organism supplementary concept word, protocol supplementary concept word, rare disease supplementary concept word, unique identifier, synonyms, population supplementary concept word, anatomy supplementary concept word] 4220

3 ('internal mammary' or 'thoracodorsal' or 'thoracoacromial' or 'lateral thoracic' or 'intercostal').mp. [mp=title, book title, abstract, original title, name of substance word, subject heading word, floating sub-heading word, keyword heading word, organism supplementary concept word, protocol supplementary concept word, rare disease supplementary concept word, unique identifier, synonyms, population supplementary concept word, anatomy supplementary concept word] 24994

4 ('free flap' or 'microsurgery' or 'DIEP' or 'ALT').mp. [mp=title, book title, abstract, original title, name of substance word, subject heading word, floating sub-heading word, keyword heading word, organism supplementary concept word, protocol supplementary concept word, rare disease supplementary concept word, unique identifier, synonyms, population supplementary concept word, anatomy supplementary concept word] 98126

5 1 and 2 and 3 and 4 100

**Web of Science**

# Web of Science Search Strategy (v0.1)

# Database: Web of Science Core Collection

# Entitlements:

- WOS.IC: 1993 to 2025

- WOS.CCR: 1985 to 2025

- WOS.SCI: 1900 to 2025

- WOS.AHCI: 1975 to 2025

- WOS.BHCI: 2008 to 2025

- WOS.BSCI: 2008 to 2025

- WOS.ESCI: 2020 to 2025

- WOS.ISTP: 1990 to 2025

- WOS.SSCI: 1956 to 2025

- WOS.ISSHP: 1990 to 2025

# Searches:

1: TS=("chest wall reconstruction" OR "breast reconstruction" OR "anterior chest wall") Results: 18098

2: TS=("recipient vessels" OR "recipient site" OR "microvascular anastomosis") Date Run: Thu Jun 26 2025 13:36:39 GMT+0400 (Gulf Standard Time) Results: 4469

3: TS=("internal mammary" OR "thoracodorsal" OR "thoracoacromial" OR "lateral thoracic" OR "intercostal") Date Run: Thu Jun 26 2025 13:36:51 GMT+0400 (Gulf Standard Time) Results: 22887

4: TS=("free flap" OR "microsurgery" OR "DIEP" OR "ALT") Date Run: Thu Jun 26 2025 13:37:13 GMT+0400 (Gulf Standard Time) Results: 90664

5: #1 AND #2 AND #3 AND #4 Date Run: Thu Jun 26 2025 13:37:32 GMT+0400 (Gulf Standard Time) Results: 216

**Ovid MEDLINE**(R) and Epub Ahead of Print, In-Process, In-Data-Review & Other Non-Indexed Citations, Daily and Versions <1946 to June 25, 2025>

1 "chest wall reconstruction".mp. or "breast reconstruction"/ or "anterior chest wall".mp. 19694

2 ("recipient vessels" or "recipient site").mp. or "microvascular anastomosis"/ 3416

3 ("internal mammary" or "thoracodorsal" or "thoracoacromial" or "lateral thoracic" or "intercostal").mp. 24991

4 "free flap".mp. or "microsurgery"/ or "DIEP".mp. or "ALT".mp. 91189

5 1 and 2 and 3 and 4 119
